# Intensive Monitoring Studies for Assessing Medicines: A Systematic Review

**DOI:** 10.3389/fmed.2019.00147

**Published:** 2019-07-19

**Authors:** Carla Torre, Maria Cary, Fábio Cardoso Borges, Paula S. Ferreira, Joana Alarcão, Hubert G. Leufkens, João Costa, Ana Paula Martins

**Affiliations:** ^1^Centre for Health Evaluation and Research (CEFAR), National Association of Pharmacies, Lisbon, Portugal; ^2^Faculty of Pharmacy, University of Lisbon, Lisbon, Portugal; ^3^Department of Epidemiology and National Cancer Registry (RON), Portuguese Institute of Oncology, Francisco Gentil, E.P.E., Lisbon, Portugal; ^4^Setubal and Santarem Regional Pharmacovigilance Unit, Lisbon, Portugal; ^5^Faculty of Medicine, Center for Evidence-Based Medicine, University of Lisbon, Lisbon, Portugal; ^6^Division of Pharmacoepidemiology and Clinical Pharmacology, Utrecht Institute for Pharmaceutical Sciences, Utrecht University, Utrecht, Netherlands; ^7^Faculty of Medicine, Institute of Molecular Medicine and Laboratory of Clinical Pharmacology and Therapeutics, University of Lisbon, Lisbon, Portugal

**Keywords:** adverse drug reaction reporting systems, clinical practice pattern, drug monitoring, pharmacovigilance, systematic review

## Abstract

**Introduction:** Intensive monitoring (IM) is one of the methods of post-marketing active surveillance based upon event monitoring, which has received interest in the current medicines regulatory landscape. For a specific period of time, IM involves primary data collection and is actively focused on gathering longitudinal information, mainly safety, since the first day of drug use.

**Objectives:** To describe IM systems and studies' data published over 11-years period (2006–2016). Specifically, we reviewed study population/event surveillance, methodological approaches, limitations, and its applications in the real-world evidence generation data.

**Methods:** We completed a systematic search of MEDLINE and EMBASE to identify studies published from 2006 to 2016, that used IM methodology. We extracted data using a standardized form and results were analyzed descriptively. The methodological quality of selected studies was assessed using the modified Downs and Black checklist.

**Results:** From 1,400 screened citations, we identified 86 papers, corresponding to 69 different studies. Seventy percent of reviewed studies corresponded to established IM systems, of which, more than half were prescription event monitoring (PEM) and modified-PEM. Among non-established IM systems, vaccines were the most common studied drugs (*n* = 14). The median cohort size ranged from 488 (hospitals) to 10,479 (PEM) patients. Patients and caregivers were the event data source in 39.1% of studies. The mean overall quality score was similar between established and non-established IM.

**Conclusions:** Over the study period, IM studies were implemented in 26 countries with different maturity levels of post-marketing surveillance systems. We identified two major limitations: only 20% of studies were conducted at hospital-level, which is a matter of concern, insofar as healthcare systems are facing a lack of access to new medicines at ambulatory care level. Additionally, IM access to data of drug exposure cohorts, either at identification or at follow-up stages, could somehow constitute a barrier, given the complexity of managerial, linkable, and privacy data issues.

## Introduction

Bridging the gap between information generated by randomized clinical trials (RCT) and how to interpret different evidence sources to better understand the real-world drug usage is of great importance, since drugs often do not perform as well in RCT as in routine clinical practice, the latter characterized by a variety of sociocultural behaviors and clinical settings (1, 2). Overtime this was clearly a lesson learned and nowadays society, including payers, demands an integrated assessment of benefits and risks under real life conditions as the next logical step after RCT (3, 4). The adoption and use of real-world evidence (RWE), defined as the clinical evidence regarding the usage and potential benefits or risks of a medical product derived from analysis of routine care data, is being increasingly important for regulatory decision-making (5, 6). RWE can provide insights into key evidentiary needs by regulators which include: (1) monitoring of medication performance in routine care, including the effectiveness, safety (e.g., labeling changes, withdrawals) and value; (2) identifying new patient strata in which a drug may have added value or unacceptable harms; and (3) monitoring targeted utilization (7).

In the last decades, a tale of withdrawals (8–10) has boosted interest in pharmacovigilance and in response, regulators have started to reform their systems, which have shifted from a largely reactive response, that relied mainly on spontaneous reporting (SR), to a more proactive approach to drug safety issues (11). Specifically, in late 2005, the US Food and Drugs Administration (FDA) and the European Medicines Agency (EMA) issued guidance documents on therapeutic risk management planning aimed at strengthening proactive postmarketing surveillance (12). More recently, the European Union implemented new pharmacovigilance legislation, where regulatory agencies have now extended powers to demand for post-authorization efficacy studies (PAES) in addition to post-authorization safety studies (PASS) (13). Overall, it has been recognized that the knowledge of drugs is no longer restricted to a binary decision at the time of marketing authorization and the prevailing paradigm changed from a risk centered approach to a benefit/risk assessment throughout the medicine entire lifecycle (1, 14).

Framed onto the scope of all these regulatory changes, intensive methods of post-marketing surveillance based on drug event monitoring (15), known as intensive monitoring (IM) methodology has been of interest (16–18). IM established systems were launched in New Zealand [Intensive Medicines Monitoring Program (IMMP)] (19) and in the UK [Prescription Event Monitoring (PEM)] (20, 21), in the late 1970s and early 1980s, respectively. Since then, these systems and its background methodology have evolved and been implemented in several geographies worldwide, such as in the Netherlands [Lareb Intensive Monitoring (LIM)] (11), Japan (22), or in some African countries (23).

As compared to SR system that passively monitors all drugs during their whole life cycle and cover all population (24, 25). IM combines the strengths of pharmacoepidemiological and clinical pharmacovigilance approaches and focuses on specific drugs. For a specific period of time, IM involves primary data collection and is defined as an observational inception cohort of subjects exposed to the drug(s) of interest (26). IM cohorts of drug exposures are identified either through prescribers (e.g., PEM), pharmacies (e.g., IMMP), and national pharmacovigilance systems (e.g., LIM) and followed in a systematic and prospective fashion through a large variety of sources (e.g., patients, prescribers, and hospitals).

Although IM systems were developed more than 30 years ago, there has not been a global comprehensive synthesis of event drug monitoring research studies to date. The purpose of this systematic review is to describe IM systems and studies' data published in the decade following the paradigm shift in medicines regulatory assessment, which was largely characterized by a more proactive approach to drug safety issues. From 2006 to 2016, we reviewed study population/event surveillance, methodological approaches (including data collection sources and analysis), limitations, main outcomes of interest, and IM applications in the real-world evidence generation data.

## Materials and Methods

This study followed current guidance of conducting and reporting systematic reviews, including guidance for undertaking reviews in health care on public health intervention reviews by the Center for Reviews and Dissemination of the University of York (27) and recommendations from the PRISMA-P statement regarding reporting items (28). The protocol for this review was registered at PROSPERO (CRD42017069309) available at https://www.crd.york.ac.uk/PROSPERO/display_record.asp?ID=CRD42017069309.

For inclusion in the review, papers had to report data on an IM study/system as defined above. RCT, studies conducted through automated databases (e.g., claims or electronic health/medical records), registries, SR schemes and case-reports/series were excluded. No restriction on study population, intervention, outcomes and comparator was imposed for study selection, although we only included studies published in English, Portuguese, Spanish, Italian, or French. Letters to editor and conference proceedings were also excluded, as these materials often reflect preliminary analysis and it is less likely that methods and results are described with the necessary details.

Electronic database identification of reports was undertaken on MEDLINE and EMBASE via OVID SP interface from inception to the 20th of April 2017, to include studies published on the time-frame of interest: January 2006 to December 2016. Complementary searches were made to identify potential additional articles: reference checking and hand-searching. The search strategy was developed after several iterations and it is presented in Additional File 1.

References located and potentially eligible for inclusion were exported to an Excel® file where authors recorded eligibility criteria of selected abstracts and full paper references. The abstracts were independently checked against the inclusion criteria by CT, MC, and PB and classified as include, unclear or exclude. The full reports for all articles that classified as include or unclear were retrieved, and two authors (CT, MC) independently evaluated its eligibility criteria for inclusion. All disagreements were resolved by discussion or, if necessary, by arbitration by a third review author (AM). The main reasons for exclusion, either at the title/abstract or at the full text screening phases were recorded.

Data from included papers were extracted by three authors (MC, CT, PB) and validated by a fourth author (FB), using a standardized data extraction form designed and pre-piloted for this review. This form was designed to systematically retrieve information from each included study on the following items: (1) general characteristics: title, first author, citation, year of publication and country, (2) type of IM system: (2.1) established systems: Cohort event monitoring (CEM), IMMP, LIM, PEM, or Modified-PEM (M-PEM) or (2.2) non-established systems/single IM studies, (3) background & rationale, (4) research question, (5) setting, (6) study design, (7) population eligibility criteria, (8) drugs studied [classified according to the Anatomical Therapeutic Chemical classification (ATC) from World Health Organization (WHO)] (29), (9) methods and data collection (variables), (10) drug domains studied, (11) data sources of events reporting [patients/caregivers (PCG), healthcare professionals (HCP)], (11) data analysis, (12) duration of follow-up and study time frame, (13) number of patients included, (14) limitations pointed by the authors, (15) authors' conclusions, (16) applications, and (17) sources of funding.

One review author (FB) assessed the risk of bias of the included studies using the modified Downs and Black assessment checklist (30), for the risk of bias and the quality of both randomized and non-randomized studies. Data was validated by another reviewer (CT) and the rationale behind assessments was documented. The Downs and Black assessment checklist was selected for the following reasons: (1) in an evaluation by Deeks et al. (31), it was one of the six instruments considered most suitable for use in systematic reviews of non-randomized studies, out of 182 tools identified; (2) it was recommended as one of the most useful tools for assessing risk of bias in non-randomized studies both by Cochrane Collaboration and the Agency for Healthcare Research and Quality (32). As some items of the Downs and Black checklist are only applicable to randomized studies and since the majority of published IM studies are a single-arm design, the Downs and Black checklist was adapted for the purpose of this review as provided in Additional File 2. Our modified checklist included a total of 13 topics out of the 27 of the original version. Consequently, the overall quality score of each study ranged between 0 and 13.

The data synthesis was descriptive as the main aim of this systematic review was to identify methods, not quantify any effect. Data from the included studies were described and presented in text, tables and figures. When multiple papers were retrieved from the same IM study (e.g., results at different follow-up periods or reporting at different outcomes/drug study domains) they were treated as a single study.

## Results

### Literature Search

**Figure 1:**
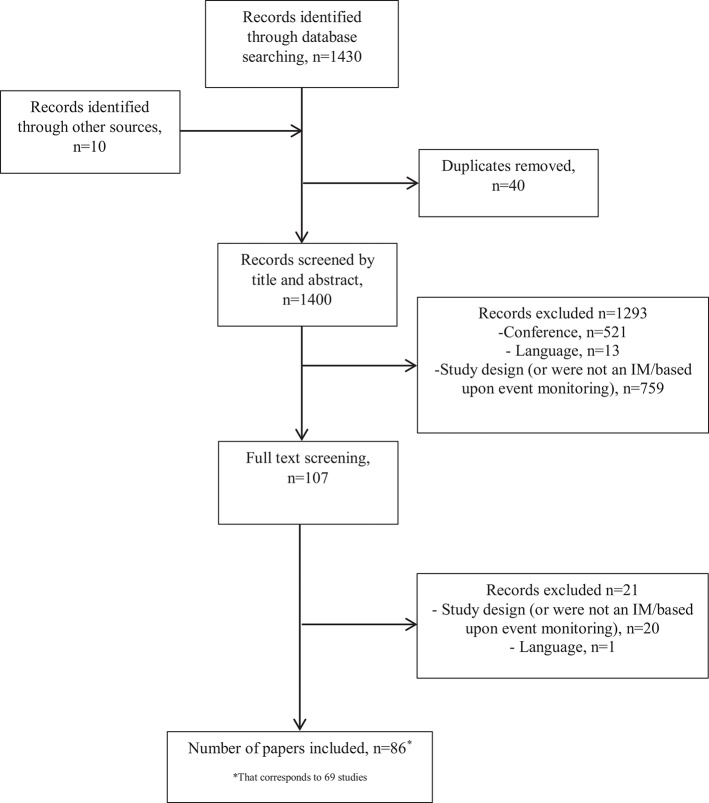
Flow chart of literature search.

The search and screening process is summarized in Figure 1. A total of 1,430 references were identified through the electronic searches of the databases. Ten additional records were identified through hand searches. After 40 duplicates were removed, we obtained 1,400 citations, which were screened by title and abstract. We excluded 1,293 citations as they did not meet the inclusion criteria, and the remaining 107 were screened full text. Twenty-one citations were further excluded (33–52) and 86 papers were included, corresponding to 69 different studies (53–138).

### Overview of Studies

The included studies were conducted in 26 countries. Overall, 70% of studies corresponded to established IM systems: PEM (*n* = 18), M-PEM (*n* = 8), CEM (*n* = 12), LIM (*n* = 6), and IMMP (*n* = 4). The remaining (*n* = 21) were single studies conducted within the IM methodology framework but were not part of any established IM. These studies were grouped in three categories: Vaccines (*n* = 14), Hospital setting based (*n* = 5), and Others (*n* = 2). Tables 1, 2 summarize the main characteristics (drugs monitored ATC, drug domains studied, event data source, methods of data collection and countries where the studies were conducted) of established and non-established IM systems. Data extracted from all included studies are presented in Table 3.

**Table 1:** Established intensive monitoring systems overall main characteristics.

	**CEM (*n* = 12)**	**IMMP (*n* = 4)**	**LIM (*n* = 6)**	**PEM (*n* = 18)/M-PEM (*n* = 8)**
Countries	BF, BY, GH, LK, MD, MZ, NG, SN, TZ	NZ	NL	UK
Methods of data collection	CEM was established by WHO. Cohort is enrolled by HCP instead of relying on prescription data supplied by pharmacies. Eligible patients are interviewed or given a questionnaire after enrolment. Patients are followed-up after a defined interval to record any new events after starting treatment with the monitored medicine. Event data is collected in medical or nursing appointments, by phone or during home visits.	IMMP operates within the NZPhvC. Patients cohorts are established from prescriptions data received from pharmacies nationwide. Questionnaires requesting information on all new events are sent to prescribing physicians (usually GP). Additional information is obtained from record linkage to other databases (e.g., deaths, hospital admissions) and SRS (reports sent by HCP, HCG and pharma companies).	LIM was developed by the national Dutch pharmacovigilance center Lareb. First time users are identified in community pharmacies (but other inclusion points are possible: e.g., GP). Baseline (registration) and event data are collected using web-based questionnaires which are sent to patients at specific time follow-up points. Data obtained reflect information from patients' perspective	PEM/M-PEM are implemented by DSRU. Patients are identified from NHS first dispensed prescriptions. Questionnaires are sent to GP to collect patient characteristics, drug exposure and event data. M-PEM differs from PEM in that a more detailed study-specific questionnaire is used (e.g., capture specific events, drug exposure, relevant disease risk factors at treatment start)
Studied domains	Safety, drug utilization patterns and effectiveness	Safety, drug utilization patterns and effectiveness	Safety and drug utilization patterns	Safety and drug utilization patterns
Setting| Event data source	AMB, HOSP| HCP, PCG	AMB| HCP	AMB| PCG	AMB| HCP
ATC	Antiinfectives for systemic use (J) and antiparasitic products, insecticides and repellents (P)	Alimentary tract and metabolism (A) and nervous system (N)	Alimentary tract and metabolism (A), antiinfectives for systemic use (J), and nervous system (N)	Alimentary tract and metabolism (A), cardiovascular system (C), genito urinary system and sex hormones (G), musculo-skeletal system (M), nervous system (N), and respiratory system (R)

*AMB, Ambulatory care level; BF, Burkina Faso; BY, Republic of Belarus; CEM, Cohort Event Monitoring; DSRU, Drug Safety Research Unit; GH, Ghana; GP, General practitioner; HOSP, Hospital care level; HCP, Healthcare Professionals; IMMP, Intensive Medicines Monitoring Programme; LIM, Lareb Intensive Monitoring; LK, Sri Lanka; MD, Madagascar; M-PEM, Modified-Prescription Event Monitoring; MZ, Mozambique; NG, Nigeria; NHS, National Health Service; NL, The Netherlands; NZ, New Zealand; NZPhvC, New Zealand Pharmacovigilance Center; PCG, Patient/Care Giver; PEM, Prescription-Event Monitoring; SN, Senegal; TZ, Tanzania; UK, United Kingdom; WHO, World Health Organization*.

**Table 2:** Non-established intensive monitoring (IM) systems: studies characteristics.

	**Vaccines (*n* = 14)**	**Hospital based (*n* = 5)**	**Others (*n* = 2)**
Countries	AU, BR, CN, FR, GT, NL, SA, TN, USA, UK	AE, GR, IT, MX, TW	FR, JP
Domains studied	Safety and effectiveness	Safety, drug utilization patterns and effectiveness	Safety and effectiveness
Setting|Event data source	AMB| HCP, PCG	HOSP| HCP, PCG	AMB| HCP
ATC	Antiinfectives for systemic use (J)	Blood and blood forming organs (B), antiinfectives for systemic use (J), antineoplastic and immunomodulating agents (L), and various (V)	Cardiovascular system (C)

*AMB, Ambulatory care level; AE, United Arab Emirates; AU, Austria; BR, Brazil; CN, China; FR, France; GR, Greece; GT, Guatemala; HOSP, Hospital care level; HCP, Healthcare Professionals; IT, Italy; JP, Japan; MX, Mexico; NL, The Netherlands; PCG, Patient/Care Giver; SA, Saudi Arabia; TN, Tunisia; TW, Taiwan; UK, United Kingdom; USA, United States of America; VAC, Vaccines*.

**Table 3:** Detailed results regarding included publications.

**IM Studies**	**ATC**	**Aim**	**Size (n); SD; DFU**	**Applications**	**Conclusions**	**QS**
**Established intensive monitoring systems**						
CEM (66)	Artemether and lumefantrine (P01BF01) and artesunate and amodiaquine (P01BF03)	Determining the safety profile of artemisinin-based combination therapies	10,259; 5; 0.2	Complement limited information from RCT. Importance of an active surveillance method in countries with low pharmacovigilance activities	Artemisinin-based combination therapies are generally safe, effective and remarkably well-tolerated among Nigerian populations	9
CEM (112)	Artemether and lumefantrine (P01BF01)	Establish the safety of artemether and lumefantrine in public health facilities in Tanzania	8,040; 34; 0.2	Complement the SRS for monitoring the safety of medicines of public health interest. CEM is a reliable pharmacovigilance tool in Tanzania	The safety profile of these drugs is favorable for the treatment of uncomplicated malaria. No major safety concerns were observed. Most of the observed AEs were already documented	9
CEM (125)	Encephalitis, Japanese, live attenuated (J07BA03)	Describe the safety profile of Japanese encephalitis vaccine in the immunization programme of Sri Lanka	3,041; 26; 1.5	Potential to identify unrecognized and unsuspected AEFI. Evidence generated strengthened the existing knowledge obtained via other studies	Life attenuated Japanese encephalitis vaccine administered at the age of 9 months is relatively safe. The AEFI were mostly non-serious	6
CEM (64)	Fixed-dose combination of dihydroartemisinin and piperaquine phosphate (P01BF05)	Assess the clinical safety of dihydroartemisinic/piperaquine in four African countries	11,028; 11; 0.9	Phase IV assessment as part of the RMP. Feasible to conduct safety monitoring of more than 10,000 patients, including electrocardiography monitoring	The treatment was well-tolerated. QT interval prolongation may occur in children	10
CEM (126)	Zidovudine (J05AF01), lamivudine (J05AF05), tenofovir (J05AF07), nevirapine (J05AG01) and efavirenz (J05AG03)	Evaluate the safety profile of the highly active antiretroviral treatment	518; 24; 12	Gather data on ADRs in resource limited settings with different populations compared with RCT (e.g., North America, Europe). Contribution patient care/therapy optimization	Achievement of a favorable benefit-risk ratio for highly active antiretroviral therapy could require more vigilant consideration and careful assessment before therapy commencement and further regular monitoring of key laboratory parameters	7
CEM (130)	Zidovudine (J05AF01), lamivudine (J05AF05) and lopinavir-ritonavir (J05AR10)	Assess the IR of AEs and adherence in occupationally-exposed healthcare workers and healthcare students	228; 78; 6	Effectively conduct an active safety monitoring of ARVs in resource limited settings where the SPRS and healthcare systems yield very little data	AE are very common and could be severe/serious in some cases. Intolerance to AE was cited as the sole reason for truncating treatment, indicating the need for effective counseling, active follow-up and AE management	9
CEM (76)	Encephalitis, Japanese, inactivated, whole virus (J07BA02)	Describe the IR and profile of overall AEs	9,798; 2; 0.5	Evidence generation on AEFI before strategizing to boost the confidence of general public on vaccination in endemic districts	IR of AEFI was several-fold higher than that reported through the national surveillance system.IR of seizures was low and vaccine related other neurological manifestations were absent	9
CEM (53, 80)	Quinine (P01BC01), pyrimethamine, combinations (P01BD51), artemisinin (P01BE01), artemether (P01BE02), artesunate (P01BE03), artemisinin and derivatives, combinations (P01BF), artemether and lumefantrine (P01BF01), artesunate and amodiaquine (P01BF03), artesunate, sulphamethopyrazine and pyrimethamine (P01BF04) and quinine and derivatives (M09AA)	Gather drug utilization and AE data for patients prescribed antimalarial medicines in an outpatient setting	2,831; 9; 0.9	Knowledge of drug utilization patterns is key in understanding patient management and consequent drug safety issues. One third of patients received an artemisinin monotherapy (not recommended by WHO due to the potential for drug resistance), highlighting the urgent need to educate health care workers for guidelines adoption. Also, improving malaria diagnostic test availability should be a priority	The first-line therapy was adhered to in <50% of cases. Captured events reflected the types of events expected and nearly all events reported are listed in the SPC of monitored medicines. Concerning drug utilization patterns, this study suggests an important role for confirmatory diagnostics in rational prescribing	11
CEM (56)	Influenza, inactivated, split virus or surface antigen (J07BB02)	Determine the distribution and types of AEs reported following immunization	5,870; 0.5; 0.2	Detection of serious event and signal generation (some reported AEs not yet included in the SPC: tachycardia, tinnitus, and decreased appetite)	The most prominent AEs reported were headaches, dizziness, muscle and joint aches, weakness, fever and injection-site pain. The types of AEFI reported were similar to other studies but the frequency of occurrence did not follow the same pattern	9
CEM (65)	Artemether and lumefantrine (P01BF01) and fixed-dose combination: artesunate and amodiaquine (P01BF03)	Determine the AEs profile of artemisinin-based combination therapies in real-life settings	3,010; 4; 0.2	Complement limited information from RCT. Importance of an active surveillance method in countries with low pharmacovigilance activities	AEs among the Nigerians were similar to those reported in the literature, including general body weaknesses, dizziness, vomiting, loss of appetite, and abdominal pain. The monitored drugs are well-tolerated among Nigerians	10
CEM (68)	Fixed-dose combination of artesunate and amodiaquine (P01BF03)	Testing CEM performance and feasibility in routine practice in malaria-endemic country	3,708; 109; 0.9	Complement limited information from RCT. Quantifying and characterizing known ADR; generating information on safety, tolerability and practical aspects	CEM-based system is feasible, but more research is needed to assess sustainability and conditions to make it cost-effective, including the amount and quality of data generated	10
CEM (78)	Sulfadoxine-pyrimethamine (P01BD51)	Evaluate the safety profile and identify potential new AEs	23,988; 8; 0.3	Simultaneous administration of sulfadoxine-pyrimethamin and routine immunizations is a safe strategy (low risk of serious AEs to infants)	Sulfadoxine-pyrimethamine is an efficient malaria control intervention with an acceptable safety profile	10
IMMP (90–92, 107)	Varenicline (N07BA03)	Describe the drug utilization of varenicline and identify ADRs (specifically psychiatry and cardiovascular events). To determine the extent of exposure during pregnancy (identify the relevant maternal and fetal outcomes)	13,176; 48; 15	Identification of a significant number of women exposed to varenicline during pregnancy. Cardiovascular events were identified (including in patients with no known history of cardiovascular disease)	Dispensing data showed that the majority of patients did not receive 12 continuous weeks of varenicline treatment as recommended. Psychiatric and cardiovascular adverse events were commonly reported in patients taking varenicline. Approximately 1% of women of reproductive age prescribed varenicline may be exposed to this medicine during pregnancy	10
IMMP (89)	Clozapine (N05AH02), olanzapine (N05AH03), quetiapine (N05AH04) and risperidone (N05AX08)	Compare nocturnal enuresis in patients taking clozapine with that in patients taking risperidone, olanzapine or quetiapine	606; 15; 15	Accurately reflect the occurrence of nocturnal enuresis in ‘real-life' use. Increased awareness of bed-wetting should lead to improved patients' care	This study showed that bed-wetting is relatively common, with about 21% of patients on clozapine (and around 10% of patients on other atypical antipsychotic medicines) likely to suffer this unpleasant and embarrassing AE	11
IMMP (88, 95)	Sibutramine (A08AA10)	Describe the patterns of sibutramine usage and to quantify the risk of fatal and non-fatal cardiovascular events	17,298; 50; 12	Complete picture of usage (monitoring of populations outside SPC: <18 years and ≥65 years)—IMMP population was younger; higher proportion of females; quantification of cardiovascular risks in general population	Extensive use of sibutramine. Some factors may have contributed to the predominantly short-term use, including costs, weight loss not meeting expectations and AEs. The risk of death from a cardiovascular event in the general population was lower than has been reported in other overweight/obese populations	10
IMMP (87)	Clozapine (N05AH02), olanzapine (N05AH03), quetiapine (N05AH04) and risperidone (N05AX08)	Investigate safety and usage of typical antipsychotic medicines in a nationwide pediatric population	420; 20; 20	Real-life picture of safety and usage of atypical antipsychotics in children. Identification of depression as a new signal for risperidone in children	Most prescriptions were for risperidone (common diagnosis: disruptive disorders). Unexpected use for sleep disorders' treatment. Depression was identified as a potential new signal for risperidone	8
LIM (77)	Metformin (A10BA02)	Gather information about frequency, latency time, outcome and management of ADRs in daily practice	2,490; 63; 12	Investigate detailed information about time course, outcome and management of ADRs to help clinicians and patients in adequate predicting and handling of drug related ADRs (improve adherence & avoid early discontinuation)	The median latency time of the most frequently reported. ADRs is <7 days. In the majority of cases, no action was taken according to metformin after the occurrence of ADRs. The findings are in line with SPC; the safety profile in daily practice is relatively safe	9
LIM (134)	Influenza, inactivated, split virus or surface antigen (J07BB02)	Evaluate the feasibility of the LIM system during the annual influenza vaccination	1,507; NM; 1	Insight into the pattern, time course, outcome, severity and possible risk factors of AEFIs compared to SRS	Intensive monitoring of AEFI is a feasible method based on willingness and possibility of participants to complete the questionnaires. The pattern of AEFI was comparable with the reported AEFI in spontaneous reports	9
LIM (115)	Varenicline (N07BA03)	Gain insight in the safety and use of varenicline in daily practice	1,418; 44; 4	Provide insight into the occurrence, latency time of ADRs and action taken with varenicline when ADRs occur. This information can be used for patients' advice (e.g., patients who start this drug)	The median reported latency times of ADRs were mostly 3–7 days, and they were mentioned with the same overall frequency as in SPC of varenicline. After experiencing nausea, abdominal pain or abnormal dreaming as possible ADRs, patients usually did not stop using varenicline	9
LIM (86)	Duloxetine (N06AX21)	Describe the user and safety profile of duloxetine in daily practice	398; 24; 6	Identification of new signals of possible new ADRs (amenorrhoea, shock-like paraesthesia, and urinary disorders)	The ADR profile of duloxetine as reported by patients is overall similar to the profile described in the SPC, but 3 new signals were identified and need further evaluation. Four patients experienced SADR (one was fatal)	9
LIM (85)	Influenza, inactivated, split virus or surface antigen (J07BB02)	Identify and quantify the AEFIs associated with the pandemic vaccine	3,569; 7; 3.2	Possibility to follow the time course of the AEFIs and to collect information about latency, recovery and duration	AEFIs due to pandemic vaccination occurred in 1/3 of participants and did not raise any concerns about the safety of vaccine. AEFIs reported were expected and non-serious	11
LIM (83, 84)	Pregabalin (N03AX16)	Gain insight into the user profile and longitudinal safety of pregabalin in daily practice	1,373; 24; 6	Contribute to improve knowledge about ADRs characteristics (IR, time course). Identification of new signals (abdominal pain, suicidal ideation and possible interaction with oral antidiabetics)	Pregabalin is a relatively safe drug in daily practice. <1.0% out of the total population experienced a serious ADR. The most frequently reported ADRs correspond to those that were most frequently reported during RCT	9
M-PEM (106)	Vildagliptin (A10BH02)	Investigate the pattern of onset and effect of vildagliptin combination therapy on peripheral oedema risk	4,828; 40; 6	Assess the occurrence of a specific AE identified within the RMP and predictors of risk	Peripheral oedema occurred most frequently within 1 month after starting treatment, and previous peripheral oedema history and male sex in elderly patients were important predictors of this risk. Concomitant use of a sulfonylurea may also increase the risk of this event	11
M-PEM (119)	Quetiapine (N05AH04)	Present a description of drug utilization characteristics	13,276; 66; 12	PASS as part of the RMP requirements: assess the long-term use (drug utilization data—determinants of prescribing and cohort characteristics) of quetiapine	The prevalence of off-label prescribing (indication and high doses) was common, as was used in special populations (e.g., very elderly). Whilst off-label use may be unavoidable in certain situations, GPs may need to re-evaluate prescribing where there may be safety concerns	10
M-PEM (105)	Fentanyl (N02AB03)	Examine the use (identify potential misuse or inappropriate/off label use) of fentanyl as prescribed in primary care	551; 32; 6	PASS as part of the RMP requirements. Feasibility of the systematic collection of physician reports of risk factors for dependence, misuse and aberrant behaviors	The prevalence of at least one pre-existing risk factor for dependence was 26%, whilst the frequency of aberrant behaviors observed during treatment was 8%. Patients with aberrant behaviors had several different characteristics to patients without	10
M-PEM (71, 100)	Varenicline (N07BA03)	Estimate the IR and the pattern of AE reported	12,159; 7; 3	Characterization of real life drug use; hypothesis testing on pre-specified events based on regulatory warnings (e.g., psychiatric and cardiovascular)	No signal was raised using the IR differences approach, and only anxiety was flagged as a potential signal. Further evaluation is needed to determine if anxiety is drug related or withdrawal symptom cause by smoking cessation	11
M-PEM (70, 138)	Rimonabant (A08AX01)	Explore possible relationships between patient characteristics and reasons for discontinuation; compare the risk of depressive episodes prior and after treatment	10,011; 24; 6	Collection of detailed information on safety issues in daily clinical practice. Assessment of risk of specific psychiatric/nervous system events of regulatory concern	Reasons for and time to discontinuation were associated with patient characteristics (e.g., medical history). Patients discontinued treatment because of psychiatric events early after starting. Regarding depressive episodes, there was no increased risk whilst taking rimonabant	11
M-PEM (75)	Modafinil (N06BA07)	Examine the safety profile of modafinil in clinical usage across a range of prescribing indications, including off-label use	1,096; 20; 6	Additional safety and characterization of real-life usage data, including in patients for whom the prescribing indication is off-label. Active surveillance post-license extension	A significant number of women of childbearing potential had not been commenced on appropriate contraceptive programmes prior to starting modafinil. The majority of events reported had been previously documented. Stratification of events according to dose revealed a number of events that occurred at the higher dose only, including serious events such as psychosis	9
M-PEM (60)	Carvedilol (C07AG02)	Investigate adherence to risk management recommendations and to evaluate the safety profile	1,666; 57; 34	Assessment of compliance with prescribing recommendations and clinical guidelines post-license extension	Regulatory guidelines were mostly followed, and most patients appeared to benefit from treatment with carvedilol for heart failure. Malaise/lassitude was the main reason for discontinuing treatment	12
M-PEM (120, 121)	Fluticasone (R03BA05) and salmeterol and fluticasone (R03AK06)	Evaluate the introduction of metered dose inhalers with new propellant into general practice	13; 413+13; 464; 19; 3	Studies conducted in response to RMP requested to manufactures (active surveillance pot-formulation change); identification of off-label use	The introduction of CFC-free inhalers was found to be generally well-tolerated	11
PEM (73)	Aliskiren (C09XA02)	Monitor the safety and use of aliskiren prescribed in the primary care setting	6,385; 40; 6	Monitoring the safety and utilization in real life setting and complementing knowledge from other sources, including SRS and clinical studies	Aliskiren is largely being prescribed for its licensed indication and is generally well-tolerated. Renal events were common in patients with risk factors for acute kidney injury	10
PEM (116, 118)	Testosterone (G03BA03)	Describe utilization characteristics and to quantify off-label use in real-life clinical practice	3,073; 48; 6	Active surveillance of drug usage in a real-life setting; identification and quantification of off-label use indications (population not included in RCT)	Only 20.9% of patients were being prescribed the monitored drug according to SPC recommendations	9
PEM (62)	Vigabatrin (N03AG04)	Compare the AE profile of children and adults taking vigabatrin, using modified SDMs	10,177; 55; 6	Contribution for assessing pediatric drug safety; provide important information to the sections of RMP linking to pediatric investigation plan; detection of differences in the safety profile (signals) between children and adults	Quantitative SDMs used together with clinical evaluation could identify possible differences in the AE profiles between children and adults	10
PEM (104)	Levocetirizine (R06AE09)	Monitor the safety of levocetirizine prescribed in the primary care setting	12,367; 19; 2	Safety analysis and characterization of real life drug use	Levocetirizine is well-tolerated when used in general practice in England. No previously unrecognized ADRs were detected	11
PEM (61)	Lamotrigine (N03AX09)	Compare AE profiles between children and adults	9,836; 44; 6	Contribution for assessing pediatric drug safety; provide important information to the sections of RMP linking to pediatric investigation plan; SDMs can be used to detect quanti/qualitative differences in AE profiles between children and adults	Differences in the AE profiles between children and adults were observed. Further, differences were observed in the proportion of ADRs reported to regulatory authorities between children and adults	11
PEM (69)	Oxcarbazepine (N03AF02)	Monitor the safety of oxcarbazepine prescribed in the primary care setting	2,243; 47; 6	Assess the safety of drugs in a real-life setting	The most frequently reported ADRs to oxcarbazepine were drowsiness/sedation, malaise/lassitude, nausea/vomiting, confusion, and rash. There were no serious ADRs reported	10
PEM (117)	Strontium ranelate (M05BX03)	Estimate the incidence of venous thromboembolism during the first 12 months of treatment	10,782; 52; 12	Contribution to the ongoing postmarketing safety assessment of strontium ranelate	The incidence of venous thromboembolism is similar to estimates in populations of similar age and corresponds to the incidence found in patients from RCT phase III and observational studies of strontium ranelate on this topic	11
PEM (81, 99, 137)	Pioglitazone (A10BG03)	Monitor the safety, describe the risk management and outcomes, and to investigate the relation between characteristics and incidence of hypoglycaemias in patients prescribed pioglitazone	12,772; 16; 8	Useful methodology for postmarketing surveillance (important pre-identified events required monitoring as part of RMP); identification of off-label use in patients with limited treatment options	Pioglitazone was considered to be reasonably well-tolerated (main reasons for discontinuing: drug not being effective). The frequency of ADRs did not exceed the frequency in SPC. Pioglitazone was associated with a low incidence of hypoglycaemia. Timely drug withdrawal and/or interventions can lead to successful resolution of class AEs	11
PEM (93, 94)	Taladafil (G04BE08)	Examine the cardiovascular safety, and to compare the mortality rate due to ischaemic heart disease in tadalafil users with that in male population	16,129; 34; 12	Assess the occurrence of a specific AE previously identified in RCT, SRS, and other post-marketing studies	Tadalafil is generally well-tolerated when used in general practice. The most frequently reported AEs were in keeping with RCT data and include headache, dyspepsia and back pain. A similar incidence of death due to ischaemic heart disease in men prescribed tadalafil to that in the male general population	12
PEM (103)	Desloratadine (R06AX27)	Monitor the safety of desloratadine prescribed in the primary care setting	11,828; 8; 6	Monitoring the safety of drugs and complement the information generated from RCT and SPR (AEs of interest)	Desloratadine is well-tolerated when used in general practice. No previously unrecognized ADRs were detected	10
PEM (74)	Esomeprazole (A02BC05)	Monitor the safety of esomeprazole prescribed in the primary care setting	11,595; 14; 6	Monitoring the safety of drugs under normal clinical practice	The safety profile of esomeprazole was consistent with the prescribing information and experience reported in the literature	11
PEM (98)	Rosuvastatin (C10AA07)	Monitor the post-marketing safety of rosuvastatin in primary care setting	11,680; 11; 6	Monitoring the safety in the real life setting and complement the information generated from other studies on specific events	Rosuvastatin was considered to be a reasonably well-tolerated drug. Abnormality of liver-function tests was found to be more frequent with the 40 mg/day dosage	10
PEM (54, 122)	Sibutramine (A08AA10) and orlistat (A08AB01)	Examine the safety profiles of sibutramine and orlistat	12,336+16,021; 25; 6	Monitoring the safety in the real life setting and complement the information generated from other studies on specific events	The AEs identified are in agreement with information from the SPC, other studies, and published case reports	11
PEM (131)	Nateglinide (A10BX03)	Examine the safety profile of nateglinide as used in general practice	4,557; 39; 6	Monitoring the safety in the real-life setting; contribute to current knowledge regarding safety during pregnancy	Nateglinide appeared to be generally well-tolerated when used in combination with metformin for the treatment of type 2 diabetes. No serious unlabelled AEs were identified	10
PEM (132)	Zafirlukast (R03DC01)	Examine the safety profile of zafirlukast as used in general practice	7,976; 37; 6	Monitoring the safety of drugs in real life setting, including population frequently excluded from RCT (patients aged <12 years) and increase knowledge in elderly where clinical experience is limited	Zafirlukast, as used in general practice, is generally well-tolerated with few associated AEs	10
PEM (109)	Apomorphine hydrochloride (G04BE07)	Examine the safety and use of apomorphine as prescribed in general practice	11,185; 21; 6	Monitoring the effectiveness and safety in the real life setting and complement the information generated from RCT on specific events	The proportion of patients for whom apomorphine was reported to be effective was low. The most frequently reported AEs were those listed in the SPC. A small number of reports for unlabelled events were thought by prescribers to be related to the drug	9
PEM (133)	Repaglinide (A10BX02)	Examine the safety of repaglinide, to quantify AE incidence and to identify previously unrecognized ADR	5,731; 32; 6	Monitoring the safety of drugs in real life setting, identification of non-compliance and increase of hypoglycaemia events with starting treatment	Repaglinide is generally well-tolerated in general practice and did not identify any serious unrecognized AEs	10
PEM (63)	Quetiapine (N05AH04)	Examine the safety profile of quetiapine as used in general practice	1,728; 37; 6	Monitoring the safety, reasons for stopping (e.g., ineffectiveness), off-label use in the real-life setting	Quetiapine is generally well-tolerated when used in general practice	10
**Non-established intensive monitoring systems**						
HOS (136)	Rituximab (L01XC02)	Evaluate the long-term safety of rituximab in rheumatoid arthritis patients in daily practice	234; 60; 27.7	Confirmation the long-term safety profile of this medicine in a refractory treatment population. Emphasizes the need for a close monitoring of treated elderly	Long-term rituximab therapy in a real-life cohort did not reveal any new safety issues. Advanced age was associated with increased risk of AEs and premature drug discontinuation	9
HOS (127)	Unfractionated heparin (B01AB01) and enoxaparin (B01AB05)	Determine the incidence of adverse outcomes in hospitalized patients	488; 13; NM	Study on population frequently excluded from RCT; identification of preventive measures (lab monitoring, dose adjustment) to reduce the risk of bleeding associated with anticoagulation therapy	Anticoagulation among hospitalized patients with CKD was significantly associated with an increased risk of bleeding and in-hospital mortality	10
HOS (57)	Rituximab (L01XC02)	Evaluate the frequency and characteristics of ADR to rituximab in patients with non-Hodgkin's lymphoma	550; 18; 0.01	Rituximab can be safely infused at a fast rate. Fast infusions can be used at hospital facilities and optimize treatment without compromising safety	Rituximab had a favorable safety profile. Contrasting with other studies, the risk of ADR was higher for slow rate infusions. The types of ADR were found to be similar with other studies, whereas the ADR incidence rate was lower	9
HOS (101)	Iopromide (V08AB05), iodixanol (V08AB09), iomeprol (V08AB10), and iobitridol (V08AB11)	Describe the nature and quantify the incidence of immediate or delayed ADRs	1,514; 15; 0.2	Identification of a “signal alarm” that recognizes anaphylaxis to contrast media as an ADR; examine predictors of immediate and delayed reactions	Both immediate and delayed ADR were of predominantly minor or moderate severity. These findings confirm that iodinated contrast media have a good safety profile.	9
					Monomeric low-osmolar contrast media bear the major responsibility as causes of immediate ADR, whereas dimeric contrast media are mainly associated with delayed	
HOS (129)	Antiinfectives for systemic use (J)	Assess the IR, risk factors, clinical manifestations and causative agents of antimicrobial-related ADR	299; 5; 0.4	Importance of clinicians being familiar with the manifestations of ADRs, since they are highly prevalent and their occurrence mimics other diseases and delay proper management	The use of antimicrobial agents caused a higher incidence of ADRs in hospitalized patients as compared with studies from western countries. Blood dyscrasias, dermatomucosal effects, and febrile reactions were the most common ADR	12
OTH (97, 111)	Fluvastatin (C10AA04)	Evaluate the long-term lipid lowering efficacy and safety of fluvastatin in Japan	21,139; 84; 60	Confirmation of efficacy and tolerability of fluvastatin; detection of a substantial impact of complications such as diabetes and hypertension or low HDL-C on cardiac and cerebral events	The results confirm the efficacy and tolerability of fluvastatin. A low risk of events in patients aged ≥65 years was found. Long-term therapy with fluvastatin elicited significantly greater improvements in lipid control in patients aged ≥65 years than in patients aged <65	10
OTH (67)	Amiodarone (C01BD01)	Assess the IR of ADRs associated with the long-term use of amiodarone and to describe their characteristics	98; 82; 38	Conduct studies among population frequently excluded from RCTs; monitoring long-term safety of drugs	During amiodarone treatment, ADRs occurred in 14 patients out of 100. Hypothyroidism, cardiac ADRs, and photosensitivity were the most frequent ADRs and occurred mainly during the first 6 months	8
VAC (110)	Influenza, influenza, live attenuated (J07BB03)	Estimate the crude IR of AEIs following vaccination with the nasal vaccine in children and adolescents	385; 3; 0.5	PASS study (European regulatory guidance on enhanced safety surveillance for seasonal influenza vaccines)	No significant change in reactogenicity or other apparent safety signal from the data collected has been detected	10
VAC (123)	Pertussis, purified antigen, combinations with toxoids (J07AJ52) and influenza, inactivated, split virus or surface antigen (J07BB02)	Measure the reactogenicity of trivalent influenza vaccine and diphtheria-tetanus-acellular pertussis vaccines administered to pregnant women	5,155; 2; 0.2	Support the safety of antenatal vaccination	Results support the safety of these vaccines administered exclusively or in combination during pregnancy, with a slight increase in mild expected ADR. Given the low incidence of systemic reactions, these results support the safety of antenatal influenza and pertussis vaccination	10
VAC (124)	Influenza, inactivated, split virus or surface antigen (J07BB02)	Implement a real-time safety monitoring program for trivalent influenza vaccine administered to pregnant women	3,173; 4.5; 0,2	Promoting confidence in vaccine uptake particularly for pregnant women; mobile phone technology proved an efficient method for timely surveillance of AEFI	Results support the safety of this vaccine in pregnant women. The low level of AEFI observed should be reassuring to antenatal patients and their providers and could be used to help promote vaccine uptake	10
VAC (58)	Diphtheria-hemophilus influenzae B-pertussis-tetanus-hepatitis B vaccine (J07CA11)	Examine patterns of clinic and emergency department visits, hospitalizations and deaths in children following vaccination	3,000; 24; 10	Capture all health care visits to monitor the safety of new vaccines in low-middle income countries	The liquid pentavalent vaccine was associated with lower rates of health care visits and not associated with increases in SAEs or hospitalizations	10
VAC (59, 102, 114)	Influenza, inactivated, split virus or surface antigen (J07BB02)	Assess the incidence and the maternal-fetal impact of 2009 influenza pandemic, and the effectiveness and the safety of maternal vaccination	877; 14; 9	IM program for pandemic vaccines (general population and pregnant women) was set up by national authority. Information on effectiveness/incidence of common AEFI of vaccination	Incidence of pandemic flu was very low in pregnant women. No effect on pregnancy and delivery outcomes was evidenced after vaccination. Seroprotection rate at delivery appeared lower than expected in vaccinated women	11
VAC (113)	Influenza, inactivated, split virus or surface antigen (J07BB02)	Assess the safety of an H1N1 vaccine during the national vaccination campaign	9,143; 8.5; 7	PASS study advised by UK medicines agency (implemented as a commitment to authorities based on European recommendations on pharmacovigilance activities)	AS03-adjuvanted H1N1 pandemic vaccine showed a clinically acceptable reactogenicity and safety profile in all age and risk groups studied	11
VAC (82)	Influenza, inactivated, split virus or surface antigen (J07BB02)	Investigate the safety of H1N1 vaccine in children and to explore the feasibility of collecting AE data through mobile telephone contacts	359; NM; 6	Feasible approach to assess the safety of medicines in developing countries, such Saudi Arabia	School-age children who received the H1N1 vaccine did not have an increased risk of hospitalization or emergency room visits. Contacting caregivers is a feasible approach to conduct studies	9
VAC (55)	Influenza, inactivated, split virus or surface antigen (J07BB02)	Estimate the frequency of AEs following vaccination against pandemic influenza A (H1N1) 2009 in children	156; 3; 1.3	Increase knowledge on special populations (children) of vaccine safety data	Systemic AEs were more frequent than local reactions at the vaccination site. IR for AEs in general and systemic reactions following the first dose were higher in children with concomitant illness or allergy. Most events were mild	12
VAC (72)	Influenza, inactivated, split virus or surface antigen (J07BB02)	Evaluate the effectiveness and safety of H1N1 vaccines	601; 1; 1	Contribute to increase information on efficacy and safety (complement the limited information generated from RCT)	The two vaccines used in Tunisia remain enough efficient to face H1N1 pandemic and are well-tolerated	9
VAC (135)	Papillomavirus (human types 16, 18) (J07BM02)	To assess the tolerability of the 2009 HPV vaccine catch-up campaign	4,248; 18; 6.2	Improve knowledge of AE to increase confidence in children vaccination; monitoring variations in rates of AE in the general population or in target group overtime	After vaccination, girls reported particularly pain at the injection site and myalgia. AE after vaccination were dose dependent (AE proportion decreased with dose) and incidence increased with age. AEs were mostly mild, and all were transient	11
VAC (108)	Influenza, inactivated, split virus or surface antigen (J07BB02)	Establish the feasibility of rapidly monitoring the new swine flu vaccines in large patient numbers receiving or offered the vaccination	4,066; 15; 7	Support the UK national strategy for H1N1 vaccine pharmacovigilance program; active surveillance tool for ‘near real-time' safety monitoring with minimal additional workload for HCP staff	No significant safety issues were identified. The use of web-based technology was successful in reducing costs and allowing the collection of high quality data directly from patients	9
VAC (128)	Smallpox vaccine (J07BX01)	Assess reported symptoms, vital status, length of hospital stay, and health-related quality of life status of vaccinated patients	203; 14; 9	Better knowledge about clinical implications of administering smallpox vaccine focusing on specific adverse cardiovascular events (complement information generated from SRS)	Although intermediate-term consequences among AEFI were not considered serious, lost days of work and a decline in health-related quality of life at the time of follow-up were common, resulting in personal economic and quality-of-life burden	7
VAC (96)	Hemophilus influenzae B, purified antigen conjugated (J07AG01), pertussis, purified antigen, combinations with toxoids (J07AJ52), pneumococcal vaccines (J07AL), tetanus toxoid, combinations with diphtheria toxoid (J07AM51), influenza vaccines (J07BB), hepatitis B, purified antigen (J07BC01), poliomyelitis vaccines (J07BF), rubella, combinations with mumps, live attenuated (J07BJ51), and varicella, live attenuated (J07BK01)	Evaluate the safety of simultaneous vaccination and the frequency of adverse reactions	772; 27; 0.2	Increase the acceptance of simultaneous vaccination	Simultaneous vaccination is feasible for Chinese applicants for a USA immigrant visa because the adverse reactions are mostly mild and temporary	9
VAC (79)	Diphtheria-hemophilus influenzae B-pertussis-tetanus-hepatitis B (J07CA11)	Document the AEFI associated with a newly introduced pentavalent vaccine in infants	406; 16; 3	Obtain information on the incidence of common AEFI of the new pentavalent vaccine; strengthen the nascent AEFI system in a resource-limited country	The results show agreement with safety studies on vaccines containing identical or similar antigens and indicate the safety and tolerability of the pentavalent vaccine in Ghanaian children	8

*ADR, Adverse Drug Reaction; AE, Adverse Event; AEFI, Adverse Event Following Immunization; CEM, Cohort Event Monitoring; DFU, Duration of Follow-up (months); GP, General Practitioners; HOS, Hospital care level; IMMP, Intensive Medicines Monitoring Programme; IM, Intensive Monitoring; IMS, Intensive Monitoring System; IR, Incidence Rate; LIM, Lareb Intensive Monitoring; M-PEM, Modified-Prescription Event Monitoring; NM, Not mentioned; OTH, Others; OQS, Overall Quality Score; PASS, Post-authorization safety study; PEM, Prescription Event Monitoring; RCT, Randomized Clinical Trial; RMP, Risk Management Plan; SADR, Serious Adverse Drug Reaction; SD, Study Duration (months); SDM, Signal Detection Methods; SPC, Summary of Product Characteristics; SRS, Spontaneous reporting system; VAC, Vaccines*.

#### Established IM Systems

PEM and M-PEM represented the majority of the studies included (*n* = 26). Concerning PEM studies, the median study duration was 35.5 months (range: 8–55) and the duration of patient follow-up varied between 2 and 12 months (median: 6.0). Similar results were found for M-PEM studies. The median number of patients *per* study was 10479.5 (range: 1,728–28,357) and 7419.5 (range: 551–26,877), for PEM and M-PEM studies, respectively. For both schemes, it was stated that all studies were conducted with unconditional funding from the pharmaceutical industry. The common limitations pointed out by the authors was the non-return by general practitioner (GP) of questionnaires (which might result in non-response bias if the characteristics of patients at responding GP practices differ from those at non-responding GP practices), under-reporting and the restriction to primary care setting. Furthermore, the lack of a concurrent control (single-group cohort design) was also addressed as a limitation, leading to a knowledge gap on the true background incidence for events. Unlike PEM, the M-PEM methodology offered a greater scope to collect information on confounding variables, since a more detailed study-specific questionnaire was used.

Considering CEM studies, the median study and patient follow-up duration, was 10.0 (range: 0.5–109) and 0.7 months (range: 0.2–12), respectively. The median cohort size was 4,789 (range: 228–23,988) patients. Five out of 12 studies were conducted with no sources of funding, 6 studies were financially supported by either governmental institutions (*n* = 3), non-governmental institutions (*n* = 2) or both (*n* = 1) and one study was financed by the pharmaceutical industry. Lack of generalizability (selection bias concerning patients' enrolment and high cohort drop-out rates), baseline events reported as “true” adverse drug events (ADE) (e.g., antimalarials studies with no event collection before vs. after treatment), costly and resource labor intensive for data collection and management were described as limitations of concern.

LIM studies reported the lowest cohort size among the established IM systems. Overall, a median number of 1462.5 (range: 398–3,569) patients were enrolled. The median study duration for the 5 out of 6 studies where this information was available, was 24 months (range: 7–63) and patients' follow-up duration varied between 1 and 12 months (median: 5.0). The majority of the LIM studies (*n* = 3) did not report the source of funding, 2 studies were conducted with financial support from governmental institutions and one was implemented without any source of funding. Limitations raised were in line with other established IM systems. LIM studies reported event rates rather than true incident rates and no information was provided about the patients that did not accept to participate (e.g., older people might be underrepresented since they do not have access/are not familiar with internet). Furthermore, since the patients were the source of event information, those who experienced an adverse drug reaction (ADR) might be more motivated to fill in a questionnaire than those who did not experience it (reporting bias). It was also stated as a limitation the difficulty in obtaining information about serious and fatal outcomes.

The median number of patients from IMMP studies was 6,891 (range: 420–17,298). The median study duration was similar to PEM studies, however a higher duration of follow-up time period (median: 15 months; range: 2–20) was observed. All studies received funding from governmental institutions and 2 studies were unconditionally co-funded by pharmaceutical industry. Not all IMMP studies reported limitations. From those studies where this information was available, an absence of a comparator group, underestimation of ADE rates and limited clinical detailed information were issues pointed out. Further, in the study of varenicline (92), the “effectiveness assessment” was performed based on information provided by the reporting doctor and for many patients, it was unknown whether varenicline was effective.

#### Non-established IM Systems

Two-thirds of non-established IM studies reported the IM of vaccines, half of those were related to the influenza H1N1 2009 pandemic vaccine. Almost all vaccines' studies (13 out of 14) targeted vulnerable populations (e.g., children, pregnant women). These studies were carried out using different methods for data collection (HCP face-to-face/web-based/telephone or mobile text messages). The median follow-up time observed was 4.5 months (range: 0.2–10) and the median study duration was 14 months; range: 1–27). The main limitations were non-response bias, non-representativeness, the lack of a control group, small sample size to detect rare outcomes (e.g., autoimmune diseases) and information bias (e.g., recall bias, adverse events following immunization (AEFI) not clinically confirmed).

IM non-established system studies classified as “Others” covered only drugs from cardiovascular system ATC main group. Regarding hospital-based studies, a wide range of drugs were monitored, although the median number of patients included was lowest (488) within all reviewed studies. Regarding funding sources, 8 out of the 21 studies did not mention the source of funding, 7 were supported by governmental institutions, 3 from the pharmaceutical industry, 1 from a non-governmental organization, and 2 reported no sources of funding.

### Overall Quality Score

The mean overall quality score (OQS) was 9.7 out of 13 (range: 7–12), being similar between established (9.9; range: 6–12) and non-established (9.7; range: 7–12) IM studies. Among established IM studies, M-PEM and PEM presented the highest mean OQS (10.5 and 10.3, respectively). Detailed results about OQS of each reviewed study are shown in Additional File 3.

## Discussion

In the decade following the paradigm shift in medicines regulatory systems, from a largely reactive response to a more proactive approach to drug safety issues (2006–2016), we thorough examined IM methodological features for data collection and analysis, population surveilled, limitations and its applications in the daily practice environment. IM studies reviewed were implemented in 26 countries with different maturity levels of post-marketing surveillance systems. IM systems operated either in countries with non-existing or weak monitoring SR schemes, such as sub-Saharan African countries (23, 139), or in countries that have the most widely used record-linkage databases in the world for drug research, such as the UK (e.g., Clinical Practice Research Datalink) (140) or the Netherlands (e.g., PHARMO) (141)—picturing the contribution of IM systems in the real-world evidence generation data. Regardless the differences found within the methodologies used, these schemes were developed with the purpose of filling the gap between RCT (high internal validity and low external validity) (142, 143), SR data (limited by under and selective reporting) (25, 144) and automated database studies (their large size and their longer follow-up times and representativeness make it possible to study real-world effectiveness and safety, but they are usually poor in detailed covariate data) (145, 146). Based on event monitoring and by tracking patients and drug use in a life-cycle based fashion, the results originating from IM studies encompasses the identification/quantification of factors that possibly negatively affect the benefit/risk balance, including (new) adverse events (identification and strengthening of signals), increase of knowledge of drug utilization patterns, identification of off-label use, among others. Moreover, by collecting longitudinal data since the first day of drug use, it allows to follow the time course (latency time and duration), outcome and management (to help clinicians and patients to adequate predicting with handling ADE, improving adherence and avoid early-discontinuation) of ADE; information that very few post-authorization methods can provide.

In the beginning of the century, Waller and Evans (147) argued that pharmacovigilance should be less focused on finding harm and more focused on extending knowledge of safety. Since then, the regulatory landscape has evolved and in parallel, an endeavor of post-marketing active surveillance schemes to meet the new regulatory challenges was witnessed. IM systems were no exception. For example, in the UK, PEM moved toward a more target surveillance: M-PEM. In the latter, efforts are done to better understand known or partially known drug risks (e.g., target analysis of events requiring special monitoring, more detailed characterization of drug usage, adherence to prescribing guidelines) and an alignment with regulatory requirements (e.g., PASS as part of RMP), is explicitly described as applications of this scheme. Further, the target sample size of 10,000 patients in conventional PEM-studies, which was driven by sensitivity assumption to detect rare and uncommon events was abandoned in M-PEM studies, where a specific sample size is calculated depending on the research question of interest (18). Some authors argue that IM is not an efficient way to detect these frequency-type events and for that purpose, other methods should be considered. For example, SR would probably be a more suitable method followed by an analytical study to confirm the signal (85). Likewise, the limited follow-up time duration does not allow for the detection of long-term events (e.g., cancer).

On the whole, drugs monitored through the reviewed studies were in the early post-marketing phase or were characterized by uncertainties concerning specific safety issues, namely those identified in the RMP (safety concerns raised from RCT, post-marketing experience and/or suspicion of inappropriate drug use). This was generally in line with IM drug entry decision criteria previously described by Coulter (19) and more recently by Harrison-Woolrych (148). Also, noteworthy that older drugs can be studied within this methodology. This was the case of metformin, marketed 60 years ago, where relevant information from the daily practice perspective, such as the outcome, management and the time course of metformin related ADE was lacking (77). We also observed that two-thirds of CEM studies were launched in resource-constrained settings and developed for monitoring artemisinin-based combination therapy for malaria treatment, aiming to complement information from RCT. In recent years, CEM was adapted and covered other drugs, such as antiretrovirals (126), vaccines (76), among others. Overtime, some practical handbooks have been issued by the WHO to support the implementation of specific programs [malaria (149), HIV/AIDS (150), and tuberculosis (151)]. The experiences of countries that have implemented CEM indicate that this was a key opportunity to raise awareness and to build pharmacovigilance capacity in these settings, which can be expected to have a positive effect on SR activities in the long run (23). The latter is of importance, since there is a need to strengthen ADR reporting rates in low-income countries and IM studies could be used in national pharmacovigilance systems (152).

Despite IM features found worldwide, the majority of monitored drugs were prescribed at the primary care level, highlighting the limited research in hospital and other secondary settings, either among established or non-established IM studies. At hospital level, where the drug market is rapidly changing, with more and more new drugs being introduced (e.g., cancer, autoimmune diseases, infectious diseases, etc.) (153), it seems that automated databases or often registries (drug registries or frequently disease registries) supplement IM systems. This might be partially due to efficiency reasons tied with decisions taken at an early stage dialogue with regulatory agencies. A recent study (154) revealed that one third of drugs approved in Europe (2007–2010), were coupled with a requirement for a registry, mainly with the purpose of gathering additional safety data. Most of the registries involved were derived from existing disease registries, i.e., designed for other purposes. The latter feature is seen as an advantage of this source due to efficiency reasons. However, it could also represent a weakness, since the multipurpose nature of registries frequently means that they are often organized for broader questions and therefore are limited by their heterogeneity in safety data collection and reporting (155). In other words, they may lack a focused hypothesis since they are viewed as a data collection structure within which studies can be performed rather than a study aimed at answering a specific research question (16, 17, 153). It is also important to cover drugs prescribed by specialists, where patients are frequently more complex in terms of underlying disease and co-morbidities. This drawback was not a reality within LIM studies, where the inclusion point was commonly the community pharmacy, but was the case of PEM/M-PEM. In the UK, to overcome this, a new IM system is being developed: the Specialist Cohort Event Monitoring (SCEM). A few SCEM studies are ongoing: OBSERVA—Observational Safety Evaluation of Asenapine and ROSE—Rivaroxaban Observational Safety Evaluation, both in response to post-authorization commitments requested by the European Medicines Agency (156).

Over the study period, the reviewed IM studies were not restricted to safety data collection. Other domains of drug outcomes, such as drug utilization patterns (both in terms of prescriber characteristics and patient population) and in a less extent, effectiveness (“therapeutic response”) were studied. Concerning safety, our review illustrated a high degree of variability and a lack of standardization. Regardless of causality assessment, terms such as “adverse event” and “adverse reaction” were often used interchangeably, without explicit definitions to ensure consistency of use. In PEM and IMMP methodology the reported information was treated as adverse events. However, in LIM studies it was stated that although a causality assessment was not performed, the term ADR was used for the reactions reported as the authors claimed that patients were asked only to report symptoms that they believed to be associated with the use of the monitored drug. In this review, we used the terms reported by the authors but we encourage developing methodological and guidance safety reporting standards, for example through scientific and collaborative working groups at international level (e.g., International Society of Pharmacovigilance and International Society for Pharmacoepidemiology).

Patients and caregivers were the event data source in 39.1% of the studies. Overtime, the evolving regulatory landscape has heightened the recognition of patients as important players in clinical practice (157). Since 2012, in the European Union, patients can report ADE directly to competent authorities. Nevertheless, the concept of patient reporting schemes is far from new—it has been around for more than 50 years (158). Studies on patient reporting have demonstrated the ability of early identification of new and strengthening potential safety signals (159–161). Moreover, reports of symptomatic non-serious ADE from PCG are of great importance, since these events are often systematically downgraded by HCP, though they play a negative role on patients' quality of life and adherence to treatment, and ultimately on the benefit-risk of a drug. On the contrary, PCG could be less valuable to detect asymptomatic or serious or fatal events (162–164).

As any other primary data collection study, IM schemes are costlier and labor intensive. In a recent survey documenting the experiences of four African countries with CEM programmes (23), limited/inadequate funding was often considered as a challenge to deal with. This constraint was also reported in the New Zealand, where due to funding cessation, IMMP was disestablished in 2013 (148). It also seems that Japan-PEM (J-PEM) is no longer operational, since no published study from this scheme was found within the timeframe of our study. The J-PEM was launched in 1997 (165) and at least two pilot studies were conducted: troglitazone (166) and losartan (167). Although, J-PEM employed the method of a concurrent-control, which represented an advantage when compared with the majority of the reviewed IM studies, it appeared to be rather complex concerning data protection and managerial issues (22).

Low response rate and/or non-response bias was frequently mentioned as a limitation of both established and non-established IM system studies. A postal survey aiming to identify reasons for non-response in PEM studies (168), found workload and lack of payment, as the main reasons for non-response. In M-PEM studies, GP were offered a modest reimbursement for completion of questionnaires, which had a positive impact on the response rate (the median response rate increased from 50% in PEM to 64% in M-PEM) (18). Moreover, unforeseen challenges when conducted CEM studies were found, namely socio-cultural reasons that led to selective/non-participation (e.g., in Kenya some women could not give informed consent without permission from their husbands) (23). In LIM studies, non-response bias was also investigated (169). The major reason for non-response raised by patients was the fact that the study was not (properly) informed in the pharmacy. Further reasons, such as time-consuming, no-access to internet or being too ill to participate, were also pointed out (170). For external validity purposes, it is important to know whether IM population is comparable to the whole population using the monitored drug. Härmark et al. (171) found that LIM population were more often male, younger and healthier (higher percentage of *de novo* treated patients, shorter disease treatment duration and less co-medication) than the reference population. The authors concluded that these differences might lead to an underestimation of events, however it was not clear whether this influenced their time-course.

Our systematic review is subject to some limitations. Firstly, unpublished research (gray literature, reports) was not captured by our search strategy and therefore not included in this study. Secondly, we acknowledge that our review is limited by what authors have reported or presented in their studies. However, an assessment of quality was performed for all reviewed studies. Despite these limitations, we believe that our results are relevant and represent the first systematic review with the most comprehensive information available of IM systems implemented worldwide.

## Conclusions

Over the study period, IM studies were implemented in 26 countries with different maturity levels of post-marketing surveillance systems, picturing the contribution of IM schemes in the real-world evidence generation data. Based on event monitoring and by tracking patients and drug use in a life-cycle based fashion, specific applications of the reviewed studies covered the following: increase of knowledge of drug safety data profile (outcome, time-course and management of ADE) identification of potential unrecognized and unsuspected ADE (tool for signal generation), gathering ADE data in resource limiting settings from populations frequently excluded from RCT (pregnant women, pediatrics and elderly), increase of knowledge of drug utilization patterns, and identification of off-label use. Overtime, an alignment with regulatory requirements was observed, where some studies have been undertaken to address specific questions related to safety concerns and drug utilization patterns (e.g., phase IV assessment as part of the RMP).

Framed onto the scope of IM systems implementation criteria, we identified two major limitations. Unexpectedly, only 20% of reviewed studies were conducted at hospital-level, which is a matter of concern, insofar as healthcare systems are facing a lack of access to new medicines at ambulatory care level (e.g., issues concerning pricing/reimbursement), and there has been a shift of new drugs introduction to hospital setting. Additionally, IM access to data of (new) drug exposure cohorts, either at identification or at follow-up stages, could somehow constitute a barrier, given the complexity of managerial, linkable and privacy data issues.

## Data Availability

All datasets analyzed for this study are included in the manuscript and/or the Supplementary Files.

## Author Contributions

MC and CT were the guarantors. All authors contributed to the study protocol, the development of the selection criteria, the risk of bias assessment strategy, and data extraction criteria. CT, MC, and JA developed the search strategy. CT, MC, and PF examined compliance of studies with eligibility criteria, with a fourth author acting as an arbiter (AM). CT, MC, and PF extracted data from reports of all included studies which was validated by a third author (FB). FB performed quality assessments which were validated by CT, with a third reviewer serving as the final arbitrator (MC). AM, HL, JA, and JC contributed to the result interpretation and discussion of results. All authors read, provided feedback and approved the final manuscript. All authors had full access to all data in the study and take responsibility for its integrity and the accuracy of the data analysis.

### Conflict of Interest Statement

CT was working at CEFAR/ANF when the study was performed and is currently employed by the Faculty of Pharmacy and has no conflict of interest to declare. MC is currently employed/receive support from CEFAR/ANF and has no conflict of interest to declare. HL reports that he is the past chairman of Dutch Medicines Evaluation Board and past-member of the EMA CHMP, and Scientific Director of the Utrecht WHO Collaborating Centre for Pharmaceutical Policy and Regulation. This centre accepts no direct funding or donations from the pharmaceutical industry or other private parties. The remaining authors declare that the research was conducted in the absence of any commercial or financial relationships that could be construed as a potential conflict of interest.
