# Supplementary material for: Intensive Monitoring Studies for Assessing Medicines: A Systematic Review
Source: Front Med (Lausanne). 2019 Jul 19;6:147. doi: 10.3389/fmed.2019.00147 (PMC6659411; doi:10.3389/fmed.2019.00147)
Supplement: Supplementary file 1 [file Data_Sheet_1.docx]

**Supplementary files**

**Additional file 1**

Search Strategy:

--------------------------------------------------------------------------------

1 Drug Monitoring/ae, mt [Adverse Effects, Methods]

2 Adverse Drug Reaction Reporting Systems/

3 (intensive adj2 monitoring).ab,ti.

4 event monitoring.ab,ti.

5 1 or 2 or 3 or 4

6 safety.ab,ti.

7 "ADR*".ab,ti.

8 (adverse adj1 (effect* or event* or outcome* or reaction*)).ab,ti.

9 6 or 7 or 8

10 exp Cohort Studies/

11 cohort$.tw.

12 epidemiologic methods/

13 controlled clinical trial.pt.

14 10 or 11 or 12 or 13

15 5 and 9 and 14

16 (meta-analysis or practice-guideline or autobiography or biography or comment or editorial or letter or news or review).pt.

17 15 not 16

18 limit 17 to yr="2006 - 2016"

19 remove duplicates from 18

**Additional file 2**

Modified Downs and Black checklist

**Reporting**

1. Is the hypothesis/aim/objective of the study clearly described?

Scores: yes=1; no=0

2. Are the main outcomes to be measured clearly described in the Introduction or Methods section?

Scores: yes=1; no=0

3. Are the characteristics of the patients included in the study clearly described?

Scores: yes=1; no=0

4. Are the interventions of interest clearly described?

Scores: yes=1; no=0

6. Are the main findings of the study clearly described?

Scores: yes=1; no=0

7. Does the study provide estimates of the random variability in the data for the main outcomes?

Scores: yes=1; no=0

9. Have the characteristics of patients lost to follow-up been described?

Scores: yes=1; no=0

**External validity**

11. Were the subjects asked to participate in the study representative of the entire population from which they were recruited?

Scores: yes=1; no=0; unable to determine=0

13. Were the staff, places, and facilities where the patients were treated, representative of the treatment the majority of patients receive?

Scores: yes=1; no=0; unable to determine=0

**Internal validity -bias**

18. Were the statistical tests used to assess the main outcomes appropriate?

Scores: yes=1; no=0; unable to determine=0

20.Were the main outcome measures used accurate (valid and reliable)?

Scores: yes=1; no=0; unable to determine=0

**Internal validity – confounding (selection bias)**

25. Was there adequate adjustment for confounding in the analyses from which the main findings were drawn?

Scores: yes=1; no=0; unable to determine=0

26. Were losses of patients to follow-up taken into account?

Scores: yes=1; no=0; unable to determine=0

**Additional file 3**

Detailed quality scores using modified Downs and Black checklist

| **IM study** | Bassi et al., 2016 | Mssusa et al., 2016 | Sanchayan et al., 2016 | Baiden et al., 2015 | Setkina et al., 2015 | Tetteh et al., 2015 | Alwis et al., 2014 | Dodoo et al., 2014/2009 | Ankrah et al., 2013 | Bassi et al., 2013 | Brasseur et al., 2012 | Sousa et al., 2012 | Harrison-Woolrych et al., 2013/2012/2011/2010 | Harrison-Woolrych et al., 2011 | Harrison-Woolrych et al, 2010; Hill et al., 2007 | Harrison-Woolrych et al., 2007 | Jong et al., 2016 | Balveren-Slingerland et al., 2015 | Oosterhuis et al., 2014 | Härmark et al., 2013a | Härmark et al., 2011a | Härmark et al., 2011b/c | Layton et al., 2016 |
| --- | --- | --- | --- | --- | --- | --- | --- | --- | --- | --- | --- | --- | --- | --- | --- | --- | --- | --- | --- | --- | --- | --- | --- |
| 1. Is the hypothesis/aim/objective of the study clearly described? | Yes | Yes | Yes | Yes | Yes | Yes | Yes | Yes | Yes | Yes | Yes | Yes | Yes | Yes | Yes | Yes | Yes | Yes | Yes | Yes | Yes | Yes | Yes |
| 2. Are the main outcomes to be measured clearly described in the Introduction or Methods section? | Yes | Yes | Yes | Yes | Yes | Yes | Yes | Yes | Yes | Yes | Yes | Yes | Yes | Yes | Yes | Yes | Yes | Yes | Yes | Yes | Yes | Yes | Yes |
| 3. Are the characteristics of the patients included in the study clearly described? | Yes | Yes | No | Yes | Yes | Yes | Yes | Yes | Yes | Yes | Yes | Yes | Yes | Yes | Yes | Yes | Yes | Yes | Yes | Yes | Yes | Yes | Yes |
| 4. Are the interventions of interest clearly described? | Yes | Yes | Yes | Yes | Yes | Yes | Yes | Yes | Yes | Yes | Yes | Yes | Yes | Yes | Yes | Yes | Yes | Yes | Yes | Yes | Yes | Yes | Yes |
| 6. Are the main findings of the study clearly described? | Yes | Yes | Yes | Yes | Yes | Yes | Yes | Yes | Yes | Yes | Yes | Yes | Yes | Yes | Yes | Yes | Yes | Yes | Yes | Yes | Yes | Yes | Yes |
| 7. Does the study provide estimates of the random variability in the data for the main outcomes? | Yes | Yes | Yes | Yes | No | Yes | Yes | Yes | Yes | Yes | Yes | Yes | Yes | Yes | Yes | No | Yes | Yes | Yes | No | Yes | Yes | Yes |
| 9. Have the characteristics of patients lost to follow-up been described? | No | No | No | No | No | No | No | No | No | No | No | No | No | No | No | No | No | No | No | Yes | Yes | No | No |
| 11. Were the subjects asked to participate in the study representative of the entire population from which they were recruited? | Yes | Yes | Yes | Yes | UD | UD | Yes | Yes | Yes | Yes | Yes | Yes | Yes | Yes | Yes | Yes | UD | UD | UD | UD | UD | UD | UD |
| 13. Were the staff, places, and facilities where the patients were treated, representative of the treatment the majority of patients receive? | UD | UD | UD | Yes | UD | UD | UD | Yes | UD | Yes | Yes | UD | Yes | Yes | Yes | Yes | Yes | UD | Yes | Yes | UD | UD | UD |
| 18. Were the statistical tests used to assess the main outcomes appropriate? | Yes | Yes | UD | Yes | Yes | Yes | Yes | Yes | Yes | Yes | Yes | Yes | Yes | Yes | Yes | Yes | Yes | Yes | Yes | Yes | Yes | Yes | Yes |
| 20. Were the main outcome measures used accurate (valid and reliable)? | Yes | Yes | UD | Yes | Yes | Yes | Yes | Yes | Yes | Yes | Yes | Yes | Yes | Yes | Yes | UD | Yes | Yes | Yes | Yes | Yes | Yes | Yes |
| 25. Was there adequate adjustment for confounding in the analyses from which the main findings were drawn? | UD | No | UD | No | No | Yes | No | Yes | No | No | No | No | No | Yes | No | No | No | Yes | No | No | Yes | Yes | No |
| 26. Were losses of patients to follow-up taken into account? | UD | UD | UD | No | UD | UD | UD | UD | UD | No | No | Yes | UD | UD | UD | UD | UD | UD | UD | UD | Yes | UD | UD |

**UD**: Unable to determine.

| **IM study** | Osborne et al., 2016 | Layton et al., 2014 | Buggy et al., 2013; Kasliwal et al., 2009 | Willemen et al., 2012; Buggy et al., 2011 | Davies et al., 2013 | Aurich-Barrera et al., 2009 | Perrio et al., 2007a/b | Coughtrie et al., 2016 | Osborne et al., 2014/2010a | Aurich-Barrera et al., 2011 | Layton et al., 2011b | Aurich-Barrera et al., 2010 | Buggy et al., 2010 | Osborne et al., 2010b | Vickova et al., 2010; Fogg et al., 2009; Kasliwal et al., 2008 | Hazell et al., 2009/2007 | Layton et al., 2009 | Davies et al., 2008 | Kasliwal et al., 2007 | Perrio et al., 2007c; Acharya et al., 2006 | Twaites et al., 2007b |
| --- | --- | --- | --- | --- | --- | --- | --- | --- | --- | --- | --- | --- | --- | --- | --- | --- | --- | --- | --- | --- | --- |
| 1. Is the hypothesis/aim/objective of the study clearly described? | Yes | Yes | Yes | Yes | Yes | Yes | Yes | Yes | Yes | Yes | Yes | Yes | Yes | Yes | Yes | Yes | Yes | Yes | Yes | Yes | Yes |
| 2. Are the main outcomes to be measured clearly described in the Introduction or Methods section? | Yes | Yes | Yes | Yes | Yes | Yes | Yes | Yes | Yes | Yes | Yes | Yes | Yes | Yes | Yes | Yes | Yes | Yes | Yes | Yes | Yes |
| 3. Are the characteristics of the patients included in the study clearly described? | Yes | Yes | Yes | Yes | Yes | Yes | Yes | Yes | Yes | Yes | Yes | Yes | Yes | Yes | Yes | Yes | Yes | Yes | Yes | Yes | Yes |
| 4. Are the interventions of interest clearly described? | Yes | Yes | Yes | Yes | Yes | Yes | Yes | Yes | Yes | Yes | Yes | Yes | Yes | Yes | Yes | Yes | Yes | Yes | Yes | Yes | Yes |
| 6. Are the main findings of the study clearly described? | Yes | Yes | Yes | Yes | Yes | Yes | Yes | Yes | Yes | Yes | Yes | Yes | Yes | Yes | Yes | Yes | Yes | Yes | Yes | Yes | Yes |
| 7. Does the study provide estimates of the random variability in the data for the main outcomes? | Yes | Yes | Yes | Yes | No | Yes | Yes | Yes | No | Yes | Yes | Yes | Yes | Yes | Yes | Yes | Yes | Yes | Yes | Yes | Yes |
| 9. Have the characteristics of patients lost to follow-up been described? | No | No | No | No | No | No | No | No | No | No | No | No | No | Yes | No | No | No | No | No | No | No |
| 11. Were the subjects asked to participate in the study representative of the entire population from which they were recruited? | Yes | Yes | Yes | Yes | Yes | Yes | Yes | Yes | Yes | Yes | Yes | Yes | Yes | Yes | Yes | Yes | Yes | Yes | Yes | Yes | Yes |
| 13. Were the staff, places, and facilities where the patients were treated, representative of the treatment the majority of patients receive? | Yes | Yes | Yes | Yes | Yes | Yes | Yes | Yes | Yes | Yes | Yes | Yes | Yes | Yes | Yes | Yes | Yes | Yes | Yes | Yes | Yes |
| 18. Were the statistical tests used to assess the main outcomes appropriate? | Yes | Yes | Yes | Yes | Yes | Yes | Yes | Yes | Yes | Yes | Yes | Yes | Yes | Yes | Yes | Yes | Yes | Yes | Yes | Yes | Yes |
| 20. Were the main outcome measures used accurate (valid and reliable)? | Yes | Yes | Yes | Yes | Yes | Yes | Yes | Yes | Yes | Yes | Yes | Yes | Yes | Yes | Yes | Yes | Yes | Yes | Yes | Yes | Yes |
| 25. Was there adequate adjustment for confounding in the analyses from which the main findings were drawn? | No | No | Yes | No | No | Yes | Yes | No | No | No | No | Yes | No | No | Yes | Yes | No | No | No | No | No |
| 26. Were losses of patients to follow-up taken into account? | UD | No | UD | Yes | No | Yes | No | UD | UD | UD | Yes | UD | No | UD | UD | Yes | UD | Yes | UD | Yes | UD |

**UD**: Unable to determine.

| **IM study** | Twaites et al., 2007a | MacLennan et al., 2006 | Marshall et al., 2006 | Twaites et al., 2006 | Vassilopoulos et al., 2016 | Sharif-Askari et al., 2014 | Arredondo-Garza et al., 2013 | Lapi et al., 2008 | Sun et al., 2008 | Morishita et al., 2012; Itakura et al., 2011 | Bongard et al., 2006 | McNaughton et al., 2016 | Regan et al., 2016) | Regan et al., 2014 | Asturias et al., 2013 | Auffret et al., 2013; Launay et al., 2012; Omon et al., 2011 | Nazareth et al., 2013 | Aljadhey et al., 2012 | Andrade et al., 2012) | Chaabane et al., 2011 | Klooster et al, 2011 |
| --- | --- | --- | --- | --- | --- | --- | --- | --- | --- | --- | --- | --- | --- | --- | --- | --- | --- | --- | --- | --- | --- |
| 1. Is the hypothesis/aim/objective of the study clearly described? | Yes | Yes | Yes | Yes | Yes | Yes | Yes | Yes | Yes | Yes | Yes | Yes | Yes | Yes | Yes | Yes | Yes | Yes | Yes | Yes | Yes |
| 2. Are the main outcomes to be measured clearly described in the Introduction or Methods section? | Yes | Yes | Yes | Yes | Yes | Yes | Yes | Yes | Yes | Yes | Yes | Yes | Yes | Yes | Yes | Yes | Yes | Yes | Yes | Yes | Yes |
| 3. Are the characteristics of the patients included in the study clearly described? | Yes | Yes | Yes | Yes | Yes | Yes | Yes | Yes | Yes | Yes | Yes | Yes | Yes | Yes | Yes | Yes | Yes | Yes | Yes | Yes | Yes |
| 4. Are the interventions of interest clearly described? | Yes | Yes | Yes | Yes | Yes | Yes | Yes | Yes | Yes | Yes | Yes | Yes | Yes | Yes | Yes | Yes | Yes | Yes | Yes | Yes | Yes |
| 6. Are the main findings of the study clearly described? | Yes | Yes | Yes | Yes | Yes | Yes | Yes | Yes | Yes | Yes | Yes | Yes | Yes | Yes | Yes | Yes | Yes | Yes | Yes | Yes | Yes |
| 7. Does the study provide estimates of the random variability in the data for the main outcomes? | Yes | No | No | No | Yes | Yes | Yes | Yes | Yes | Yes | Yes | Yes | Yes | Yes | Yes | Yes | Yes | Yes | Yes | Yes | Yes |
| 9. Have the characteristics of patients lost to follow-up been described? | No | No | No | No | No | No | No | No | Yes | No | No | No | No | No | No | Yes | No | No | Yes | No | No |
| 11. Were the subjects asked to participate in the study representative of the entire population from which they were recruited? | Yes | Yes | Yes | Yes | UD | Yes | Yes | UD | Yes | Yes | UD | Yes | Yes | Yes | Yes | Yes | Yes | UD | Yes | UD | Yes |
| 13. Were the staff, places, and facilities where the patients were treated, representative of the treatment the majority of patients receive? | Yes | Yes | Yes | Yes | UD | Yes | UD | UD | UD | Yes | UD | Yes | UD | Yes | Yes | UD | Yes | UD | UD | UD | UD |
| 18. Were the statistical tests used to assess the main outcomes appropriate? | Yes | Yes | Yes | Yes | Yes | Yes | Yes | Yes | Yes | Yes | Yes | Yes | Yes | Yes | Yes | Yes | Yes | Yes | Yes | Yes | Yes |
| 20. Were the main outcome measures used accurate (valid and reliable)? | Yes | Yes | Yes | Yes | Yes | Yes | Yes | Yes | Yes | Yes | Yes | Yes | Yes | Yes | Yes | Yes | Yes | Yes | Yes | Yes | Yes |
| 25. Was there adequate adjustment for confounding in the analyses from which the main findings were drawn? | No | No | No | No | Yes | No | No | Yes | Yes | No | No | No | Yes | No | No | No | No | No | Yes | Yes | Yes |
| 26. Were losses of patients to follow-up taken into account? | UD | UD | UD | UD | UD | No | UD | UD | Yes | UD | UD | UD | UD | No | No | Yes | Yes | Yes | Yes | UD | Yes |

**UD**: Unable to determine.

| **IM study** | Mackenzie et al., 2011 | Sniadack et al., 2008 | Hua et al., 2008 | Dodoo et al., 2007 |
| --- | --- | --- | --- | --- |
| 1. Is the hypothesis/aim/objective of the study clearly described? | Yes | Yes | Yes | Yes |
| 2. Are the main outcomes to be measured clearly described in the Introduction or Methods section? | Yes | Yes | Yes | Yes |
| 3. Are the characteristics of the patients included in the study clearly described? | Yes | Yes | Yes | Yes |
| 4. Are the interventions of interest clearly described? | Yes | Yes | Yes | Yes |
| 6. Are the main findings of the study clearly described? | Yes | Yes | Yes | Yes |
| 7. Does the study provide estimates of the random variability in the data for the main outcomes? | Yes | No | Yes | No |
| 9. Have the characteristics of patients lost to follow-up been described? | No | No | No | No |
| 11. Were the subjects asked to participate in the study representative of the entire population from which they were recruited? | Yes | UD | UD | Yes |
| 13. Were the staff, places, and facilities where the patients were treated, representative of the treatment the majority of patients receive? | UD | UD | UD | UD |
| 18. Were the statistical tests used to assess the main outcomes appropriate? | Yes | Yes | Yes | Yes |
| 20. Were the main outcome measures used accurate (valid and reliable)? | Yes | Yes | Yes | Yes |
| 25. Was there adequate adjustment for confounding in the analyses from which the main findings were drawn? | No | No | Yes | No |
| 26. Were losses of patients to follow-up taken into account? | UD | UD | UD | UD |

**UD**: Unable to determine.
